# Voluntary Work and the Voluntary Hospitals

**Published:** 1910-10-01

**Authors:** 


### SPECIAL INSTITUTIONAL ARTICLES.

### Voluntary Work and the Voluntary Hospitals.

EMINENT CHAIRMAN SERIES.

The present position of the voluntary hospitals, which is stronger financially and better administratively on the whole than it has ever been before in the history of voluntary institutions, is due largely to the increased number of men of position who have, during the last fifteen or twenty years, devoted much of their leisure and energies to the cause of the sick. Whatever may be said for and against the claims of the members of the honorary medical staff of voluntary hospitals to public recognition for the services they render to the sick, no one can question the fact that a layman of eminence who takes high office and works zealously for years in a voluntary hospital as treasurer or chairman is entitled to public thanks and recognition for the important voluntary services he thus renders in the cause of the sick. We propose to give, once a month, the portrait and a historical account of the work done by some of the ablest and greatest chairmen and treasurers who have been associated with the progress of great voluntary hospitals. In this way we hope to do justice to the individual, and at the same time to encourage others to engage in this important work, by showing how essential it is to the prosperity and progressive development of a great voluntary hospital that men of influence and devotion should give largely of their time and influence to the cause of the hospitals.

### Hospital Families.

Formerly the county and many other voluntary hospitals depended mainly for their vitality and means upon the support and influence of a few men and women and their families and friends. The Buxtons have been honourably associated for generations with the work, development, extension, and maintenance of the London Hospital, to mention only one instance. Such services were invaluable; but they were liable to the discount that, doing so much for a given hospital, the public was inclined to think that it was so fortunately situated that further outside help and fresh blood were rarely necessary. People were apt to persuade themselves too that the proffer of such help might be resented by the few who had devoted so much time and money to the support of a particular hospital. So, like most other good things in this unequal world, the family support of the few was open to criticism and misunderstanding. Hence the establishment of King Edward's Hospital Fund and the League of Mercy came as a very real blessing to many hospitals.

### The Royal Example.

King Edward's Hospital Fund has brought much money and the enforcement of wise principles of economy to the Metropolitan hospitals directly. It has indirectly resulted in great good in similar ways to voluntary hospitals all over the Empire. The League of Mercy has recruited an army of workers in the cause of the same group of hospitals, and has extended the area of givers in all ranks of society to an extent which has done much to consolidate and secure the voluntary system of monetary aid. But, apart from and beyond these direct benefits, the example set by King Edward VII., by King George V., and Queen Mary, with other members of the Royal Family, who have deliberately set themselves to give, regularly every year, a large measure of personal service in the cause of the sick poor, has produced a change in public opinion, and made many people, who otherwise might have never given themselves, their time, and money to the voluntary hospitals, follow this precedent. So far-reaching has the Royal example been that we have found large private firms, the partners of which are amongst the busiest and hardest-worked men in the world, where every partner had identified himself with some one hospital, to the interests of which he has devoted much of his limited leisure for years past.

### Wanted—More Men.

The need for men of this type in hospital work was never so apparent as it has become in recent years. We are threatened with a social upheaval of which no one can forecast the end with any certainty. It is therefore of paramount importance, seeing that the voluntary hospitals as a whole are probably better managed than any other type of hospital in the world, that the ablest and most active minds in the business world should devote the best they have to give to the hospitals. The papers read at the British Hospitals Association Conference at Glasgow this week demonstrate how diverse are the views and how complicated are the issues raised by a consideration of the present and future organisation of an adequate service of medical and hospital aid for the sick of all classes. Mr. Loch would rely, it would seem, upon the spontaneous and voluntary combination of voluntary agencies, leaving the Government service to supply the demands for medical aid by the poor and improvident. Mrs. Sydney Webb, on the other hand, is evidently in favour of distributing medical aid and comforts with hospital and institutional care by the old means of the bellman sent out into the highways and byways to beg all and sundry to choose for themselves and their families which of these various advantages they consider themselves in need of, and which they will accept as their right at the hands of a fraternal movement. There is great need for the wise head and controlling hand of a statesman of experience and knowledge to take in hand the reports of the Poor Law Commission, and to weave a practical working scheme on a co-operative basis to secure for the sick of all classes the medical aid they may need, upon a system which preserves the independence and character of the hospital patient, whilst it offers him full opportunity to pay, when able, according to his means, for the treatment he requires when overtaken by illness or accident. We hope to return to this branch of the subject shortly, and meanwhile we welcome the discussions and papers at the Glasgow Hospital Conference as helpful to a better understanding of the issues raised.

### A Biographical Series.

Reverting to the series of articles upon great hospital treasurers and chairmen, we desire to say that these papers will be written in the public interest, with the hope that every great voluntary hospital's work may thus become better known and appreciated by those who sympathise with the sick and are interested in their claims upon the healthy and prosperous. We have the pleasure to express our acknowledgments to many busy workers in the cause of the voluntary hospitals who have promised to help to make this series of articles as interesting, helpful, and beneficial to the hospitals as possible. We wish to make it clear that the purpose of each article will be not to bring the individual worker into great prominence at the expense of his hospital, but always to put the hospital and its interests first. We hope at the same time to do justice to every worker who by his devotion has contributed largely to the success of a particular hospital. We entirely sympathise with and respect the feeling which makes the true hospital worker regard what he does for his hospital as a high privilege and sacred duty. All our best and most successful hospital workers have exhibited this spirit and displayed their honourable feelings. Each sketch will therefore be written with the object of making the valuable work of each great hospital known and appreciated, and not primarily, from the point of view of personal journalism which makes the individual so prominent that the principles which may underlie his work are relegated to the background. Our object then, whilst doing full justice to the individual, will not be to make the chairman known because of his work for the hospital, but to make the hospital, if possible, better known and appreciated because of the services rendered to the institution by each chairman or treasurer and his friends. We should, therefore, welcome the co-operation of any one associated with the voluntary hospitals who can send information or a sketch of the work of any big-hearted devoted layman who has done much for his hospital without any thought of the personal cost at which such services have been rendered.

